# FDA’s Nozzle Numerical Simulation Challenge: Non-Newtonian Fluid Effects and Blood Damage

**DOI:** 10.1371/journal.pone.0092638

**Published:** 2014-03-25

**Authors:** Miquel Trias, Antonio Arbona, Joan Massó, Borja Miñano, Carles Bona

**Affiliations:** The Institute of Applied Computing & Community Code (IAC^3^), University of the Balearic Islands, Palma, Spain; University of Arizona, United States of America

## Abstract

Data from FDA’s nozzle challenge–a study to assess the suitability of simulating fluid flow in an idealized medical device–is used to validate the simulations obtained from a numerical, finite-differences code. Various physiological indicators are computed and compared with experimental data from three different laboratories, getting a very good agreement. Special care is taken with the derivation of blood damage (hemolysis). The paper is focused on the laminar regime, in order to investigate non-Newtonian effects (non-constant fluid viscosity). The code can deal with these effects with just a small extra computational cost, improving Newtonian estimations up to a ten percent. The relevance of non-Newtonian effects for hemolysis parameters is discussed.

## Introduction

Computational Fluid Dynamics (CFD) is commonly used by engineering teams in the design and development of many cardiovascular medical devices. However, its applicability for both demonstrating device safety and predicting potential problems based on patient-specific data is still very limited. In most cases, the efficacy of these techniques has not been fully proven.

The main reason inhibiting the use of computational methods for such purposes within a regulatory review is the lack of reliable standarized methods. In order to meet this need, the U.S. Food and Drug Administration (FDA) recently completed a computational interlaboratory study [1] to determine the suitability and methodology for simulating fluid flow in an idealized medical device. In particular, the goal of the FDA’s challenge was to establish a set of experimentally validated benchmark computational models applicable to cardiovascular medical devices. As the FDA’s challenge article [1] states: “It is imperative to undertake and openly publish high quality validation cases relevant to the biomedical community (for both fluid dynamics and blood damage) to help improve the quality of biomedical CFD simulations”.

The FDA’s CFD challenge covers a whole range of Reynolds numbers, involving different physiological scenarios and moments of the cardiac cycle. This paper focuses on the low Reynolds (laminar) regime. We intend to contribute to the question of whether in such regime the simpler Newtonian models are enough or if, on the contrary, one must consider non-Newtonian models from the start. Non-Newtonian models are only potentially relevant in the laminar regime, as their effects tend to diminish with increasing Reynolds number.

Different regimes will appear at different moments of the cardiac cycle and in different locations. Therefore a single code aiming at simulating full cardiac cycles, or dealing with devices that create around them different regimes should handle both turbulent, transitional, and laminar regimes. The development of such codes is far from trivial. Usually, each regime is targeted with quite different physical models (variations of the basic Navier-Stokes equations) and discretization techniques. If, additionally, non-Newtonian models are an ingredient we need to fit in, the whole picture is even a bit more complex.

We intend to develop one of such codes, with an incremental strategy. In this sense, our approach consists in taking as starting point a Direct Numerical Simulation (DNS) approach for the laminar regime, with a view to extend later its validity to the transitional regime by means of either adopting Adaptive Mesh Refinement (AMR) capabilities or by including models used in Large Eddy Simulations (LES) when faced with the transition to turbulence. Our simulation code was automatically generated by Simflowny [2], a general-purpose platform for the management of physical models and simulation problems developed by ourselves at the 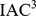.

In this first paper, we address the laminar regime, limiting ourselves to the proposed 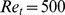 case. This allows us to investigate the role of non-Newtonian effects (non-constant viscosity). Even if we confirm previous claims [3], [4] limiting the non-Newtonian effect in physiological parameters up to a ten percent, let us note that our code can incorporate such effects with virtually no computational cost. In this sense, we could also say that we are improving the results up to a ten percent without a significant extra computational cost. On the other hand, our results point out that even a few percent on the flow variables can translate into something bigger in crucial parameters, such as blood damage.

A side result of this paper is to provide a way of computing blood damage, both accurate and reliable. A novel analytical expression setting a lower limit on blood damage in axisymmetric problems is also given. Using this expression as a reference level,the role of non-Newtonian effects in blood damage cannot be disregarded.

This article is organized as follows. In section Materials and Methods we summarize the main details of the FDA’s CFD challenge, we describe the physical models we used in CFD simulations, with details of our implementation, and we discuss the physiological parameters under consideration, focusing particularly on hemolysis. Section Results and Discussion presents our results in comparison with the ones collected by the FDA study. Finally, section Conclusions summarizes our main conclusions.

## Materials and Methods

### FDA’s Nozzle Challenge

**Figure 1:**
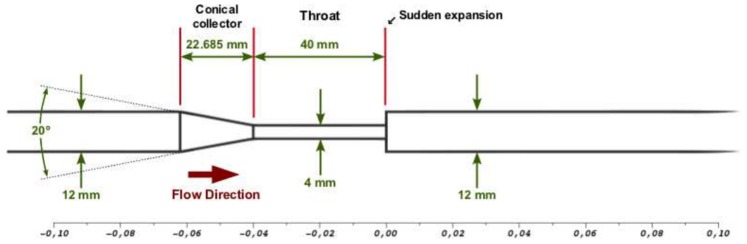
Nozzle geometrical specifications defined in the FDA CFD challenge.

The FDA interlaboratory study [1] considered a simplified, idealized medical device consisting of a small nozzle, which shares characteristics of blood-carrying medical devices, such as blood tubing, hemodialysis sets, catheters, cannulas, syringes and hypodermic needles. The device was designed to include accelerating flow, decelerating flow, variations in shear stress and velocities, and recirculating flow, all of which may be related to blood damage in medical devices. The model geometry was a 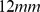 diameter cylindrical nozzle with a 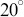 conical collector and sudden expansion on either side of a 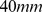 long, 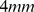 diameter throat. Figure 1 schematically represents this geometry.

Three laboratories generated experimental validation data on geometrically similar physical models using particle image velocimetry (PIV) techniques. Concurrently, twenty-eight groups from around the world submitted simulation results for five flow rates, spanning laminar, transitional and turbulent flows. The simulations showed considerable variation from each other and from experiments. Both experimental and submitted CFD results are available at https://fdacfd.nci.nih.gov/.

Data available from the experimental data sets are: (a) distributions along the nozzle’s centerline of pressure, axial velocity and Reynolds stress; (b) profiles along radial cuts at different positions of the nozzle of axial velocity, radial velocity, shear stress and Reynolds stress; (c) wall distribution pressure and Wall Shear Stress (WSS); and (d) jet width along the centerline. Shear rates were indirectly calculated from the velocity field (assuming some viscosity model) and pressures were also measured in a separate acrylic model manufactured with wall pressure taps along the length of the nozzle.

Five different flow rates were proposed, corresponding to throat Reynolds numbers of 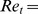
{500, 2000, 3500, 5000, 6500}, spanning from the laminar regime to turbulence, including transitional flows. In this article, we will focus on the lowest 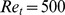, corresponding to a laminar regime, in which DNS techniques are applicable and also where lower shear rates are found and therefore non-Newtonian effects become more relevant. The DNS approach minimizes the role of phenomenological assumptions, and therefore decreases the risk that a conclusion on the relevance of non-Newtonian effects on blood damaged is affected by errors due to these approximations. This throat Reynolds number represents [1] an inlet Reynolds number of 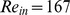, which corresponds to a flow rate of 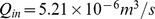.

### Numerical Scheme

The model we use in order to characterize the hemodynamics in the FDA’s scenario is given by the Navier-Stokes equations [5], [6], a set of PDEs for the time evolution of density, 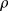, and linear momentum, 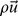, fields.

*Continuity equation:*

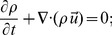
(1)


*Momentum equation:*

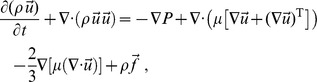
where 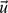 is the velocity field, 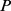 the hydrodynamic pressure, 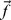 an external force, 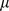 the viscosity coefficient, and superscript ‘
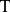
’ means (matrix) transpose. Equations (1) and (2) describe compressible, viscous (even with non-uniform 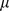) fluids; the only assumption we made is neglecting the bulk viscous term [6].

In comparison with the simpler Euler equations for ideal fluids, Navier-Stokes equations add the possibility of including the viscosity of the fluid. In the particular case of hemodynamics, viscosity plays an important role; moreover, the viscosity coefficient cannot always be assumed to be constant, as blood actually has a non-Newtonian behavior.

In addition to the Navier-Stokes evolution equations, one must also provide the equation of state that relates the pressure with the remaining fields. In many CFD blood flow simulations, the incompressible fluid approximation is adopted, converting the conservation of mass (evolution) equation, Eq. (1), into a constraint equation. The equation of state makes no sense in this approach, but still one must provide a prescription for the pressure distribution. We will rather adopt a different strategy by considering a slightly compressible model of blood [7] –all that is required is to choose a speed of sound large enough for the density fluctuations to be negligible.

The physical scenario consists in a laminar fluid through an axisymmetric nozzle. Using cylindrical coordinates, axial symmetry can be invoked for reducing the number of required variables, by neglecting any angular dependency. All field dependencies are then written in terms of radial and axial coordinates, 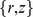, and vectors are decomposed into components in such two directions. This 2D simulation misses some features of the 3D one, such as asymmetric flow instabilities, but allows a much efficient use of computational resources.

In order to numerically solve the evolution equations, we discretize them over a regular mesh and use finite-difference methods to compute spatial derivatives. In particular, we have used a high-resolution finite-difference scheme based on the Osher-Chakrabarthy family of linear flux-modification schemes [8], [9], combining a 7th order algorithm in the fluid interior cells with a 3rd order method near the boundaries. The numerical code used to perform our simulations was automatically generated by Simflowny [2], a general-purpose platform for the management of physical models and simulation problems developed at the 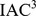. The resulting code is written in C++ and built over an extensible simulation framework, SAMRAI [10], which allows for parallelization on distributed-memory clusters and adds the possibility of using AMR. The results presented in this article correspond to a relatively low Reynolds number, 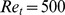, so they didn’t require such AMR capabilities. We obtained them by running on a small size, 32-CPU, computer cluster. A convergence test, checking mass conservation, is presented in Appendix S1.

Our code implements non-reflecting characteristic inflow and outflow boundary conditions [11]. Outflow conditions require to set a reference pressure value (or density, through the equation of state), e.g. 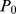, that would correspond to the arterial pressure of the patient in case of implanted medical devices, leaving free (fixed by the evolution itself) the velocity values. Reciprocally, inflow conditions require to set an inflow velocity instead of pressure or density values. In our simulation, the inflow boundary is considered to be far enough from the conical collector and throat regions so that an inlet Poiseuille flow can be assumed. This is,
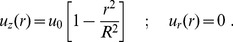
(2)where 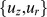 are the cylindrical velocity components, 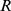 is the radius and 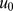 is the axial velocity at the nozzle centerline. The latter can be easily related to the incoming volumetric flow rate by integrating the axial velocity field over the transverse section (see Eq. (6)), resulting into 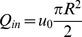.

### Equation of State: Slightly Compressible Blood

For the purposes of physiological modelling, it is very important to obtain the pressure distribution. In many hemodynamic simulations, blood is assumed to be an ideal incompressible fluid, which replaces the (dynamic) continuity equation by a kinematic constraint on the velocity field, besides decoupling density and pressure (no equation of state required). This simplifies the set of evolution equations; however, it adds the difficulty of obtaining the pressure profile [12], especially when working with finite-difference or finite-volume methods, which require the use of ad-hoc implicit algorithms such as SIMPLE [13] or PISO [14].

An alternative approach is to consider slightly compressible fluids, where one may use the continuity equation to determine the density, and the pressure is calculated from an equation of state. All what is required is that the speed of sound is large enough for the density fluctuations to be negligible. Notice, for instance considering equation of state (3), that density fluctuations 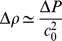, with 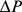 being pressure variations due to actual gradients. The equation of state most frequently used is due to [7], which is also the equation of state used in most Smoothed Particle Hydrodynamics (SPH) applications [15]:
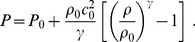
(3)


The previous equation is characterized by four parameters: a reference pressure, 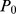, the fluid’s density, 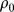, the speed of sound 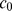 at that reference pressure and the adiabatic index 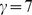. In our case, following FDA’s specifications, blood is modelled with 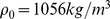. Results are independent of the value of the reference pressure (what really matters is the pressure gradient), but anyway the selected value is 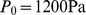. Finally, we have checked that, al least for this particular problem, using a reduced sound speed 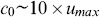 gives the same results as using the (much higher) actual value; in particular we routinely use 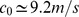. The numerical stability of our explicit finite-difference scheme would force us to reduce dramatically the size of the time step if we took the real value, resulting in a prohibitively long simulation time without actually changing the outcome results in a significant way.

With this blood model, the resulting fluid is just slightly compressible. Indeed, we have observed that the resulting density fluctuations are actually smaller than 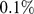. On the other hand, the benefits are that pressure values are obtained directly as an evolution field. The slightly compressible fluid model requires the use of non-reflecting boundary conditions, otherwise any small shock wave originated even at the early stages of the evolution (for instance, due to the initial conditions) will not leave the computational domain, distorting the whole simulation after a short time. Such non-reflecting boundary conditions are obtained by making use of the characteristic decomposition of the evolution equations, in particular, by treating differently the incoming and outgoing information through the boundaries [11].

### Newtonian vs Non-Newtonian Rheological Models

Blood is a complex fluid [16]. Although it is frequently modelled as water-like (as an incompressible Newtonian fluid), it is more properly described as a pseudoplastic (as lava, ketchup or paint) when non-Newtonian effects are properly taken into account [17]. Actually the study of rheological properties of blood is still subject to rapid advance, particularly regarding its in-vivo properties.

#### Carreau-Yasuda viscosity model

A realistic and quite simple model of non-Newtonian effects on blood viscosity is the Carreau-Yasuda (CY) model. This model takes into account the rise of blood viscosity at low shear rates, including a smooth transition function between the high viscosity value at low shear rates and the low one at high shear rates [17]. In particular, the viscosity model reads as follows:
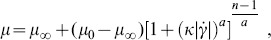
(4)where 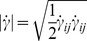 is the effective shear rate; 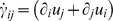 is the shear rate tensor, 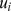 being the velocity field; 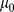 and 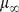 are the viscosities at zero and infinity shear rates; and 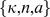 are constant parameters. Using calibrated results for blood from [18], we have that: 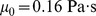, 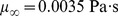, 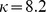
s, 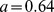, and 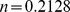.

**Figure 2:**
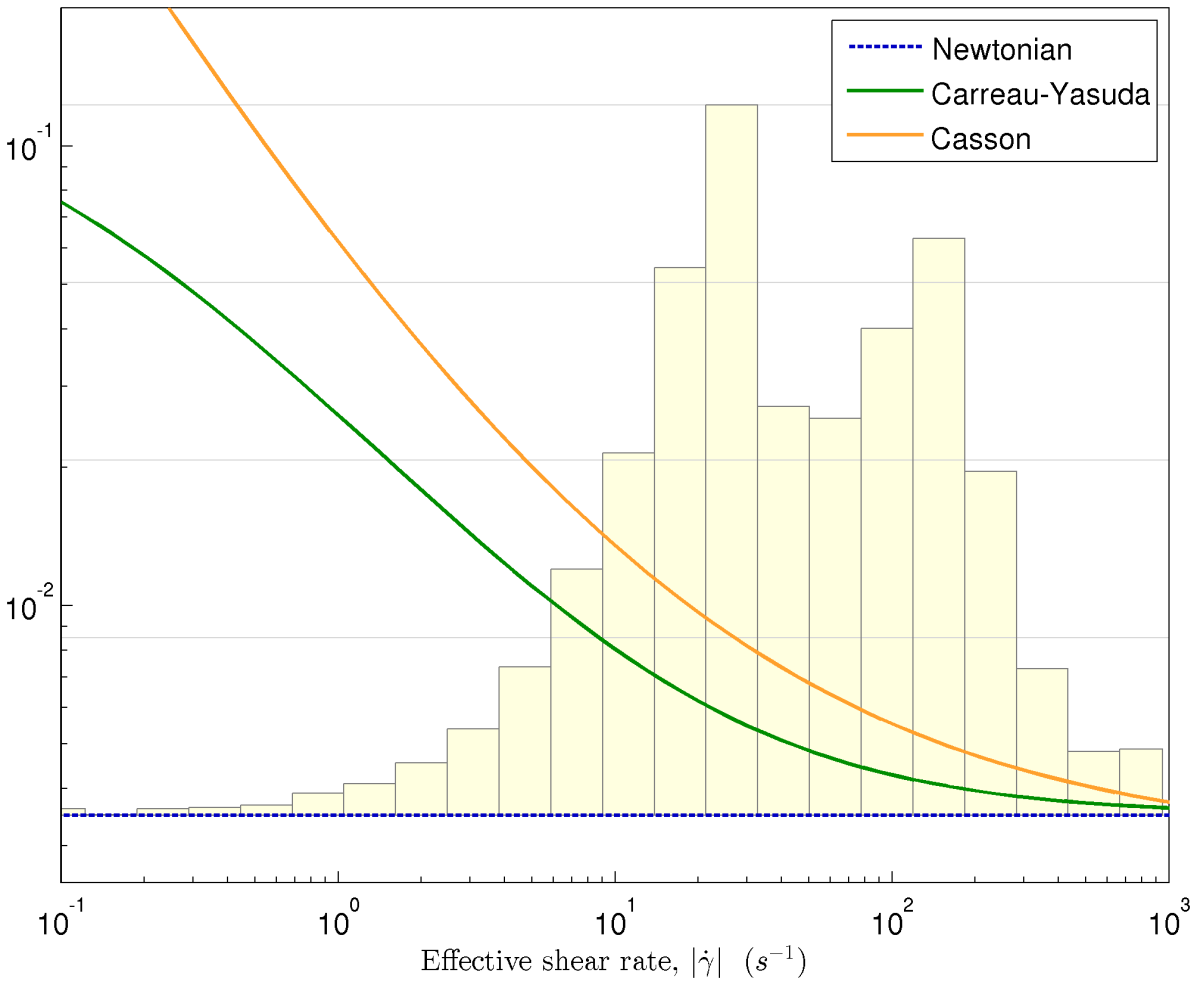
Curves: blood viscosity values as a function of the effective shear rate for different rheological models. Histogram: actual distribution of shear rates in the CFD simulation results presented in section Results and Discussion.

Actually, the Newtonian approximation consists in using a constant viscosity value, equal to 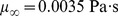. Figure 2 plots blood viscosity as a function of the effective shear rate using the different rheological models considered in this article.

#### Casson viscosity model

Another widely used alternative to describe the non-Newtonian behavior of blood is the Casson model. In this model the apparent viscosity is given by [17]:
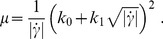
(5)


The effective shear rate, 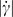, is the same quantity as previously defined in the CY subsection. In general, the two parameters 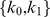 are functions of the hematocrit count [19]. In this article, we consider parameter values of 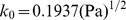 and 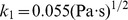, obtained from [19].

The Casson model fits empirical data quite well for shear rates of 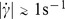. However, it provides unrealistic (divergent) viscosity values at very low shear rates (see Figure 2). For these low shear rates, we have implemented a cutoff model [17] where for values of 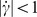, we utilize the value of 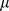 corresponding to 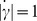.

### Fluid-solid Interaction

We treat walls as a static, non-deformable, rigid body. Fluid interaction comes through the no-slip condition for viscous fluids, which states that at a solid boundary, the fluid will have zero velocity relative to the wall. The density and pressure fields have to be adjusted accordingly. In particular, we must use the characteristic decomposition of the Navier-Stokes equations in order to discern between the incoming and outgoing characteristic fields, and only alter the incoming ones in such a way that the no-slip condition is being satisfied [20]. This solution is also the one being used in general fluid-solid interaction, where, additionally, a balance force between fluid and solid must also be considered.

### Relevant Physiological Indicators

#### Conservation of mass metric

There are some fundamental conservation laws that CFD simulations should obey, and conservation of mass is one of them. In case one considers incompressible flows, the conservation of mass becomes equivalent to the conservation of volumetric flow rate, 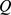, which can be obtained by integrating the velocity over a nozzle transverse section:
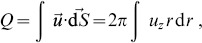
(6)which applied to a Poiseuille inflow would translate into 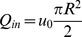.

For slightly compressible flows it is more exact to actually compute the mass flow rate as the transversal integral of 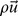, this is: 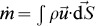, which for a Poiseuille flow turns out to be.
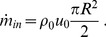
(7)


The mass flow rate is not a medically relevant physiological indicator *‘per se’* since it is set by the inflow boundary condition. Nonetheless, it is a very useful indicator of the quality of a CFD simulation, since it should be conserved along the fluid path.

#### Pressure drop

Pressure recovery, given in Pascal units or mm of Hg, provides an indicator of the blood’s energy loss when going through a valve or a device, both due to the narrowing and turbulent effects. In cardiac applications, the pressure drop also indicates the heart’s effort in pumping blood. Pressure drop values can be measured or obtained in several different and independent ways:

- At the FDA’s laboratory experiments, pressure values were measured in a separate acrylic model manufactured with wall pressure taps along the length of the nozzle.
- From CFD, simulation pressure can be obtained as an independent field if one is using a slightly compressible model, like in this article; or from the velocity field if working under the incompressible assumption.
- Clinically, with real patients, the pressure drop along a valve or device cannot be directly measured. Instead, physicians make use of the simplified Bernoulli equation to non-invasively estimate the pressure drop at the narrowing from velocity measures obtained using Doppler techniques [21], [22]. Bernoulli’s principle states that the sum of all forms of mechanical energy in a fluid is constant along a streamline. This result is valid under the assumptions of non-viscous, steady flow. The widely used simplified Bernoulli equation [21] is applied in these cases along the streamline that follows the centerline of the artery, assuming that the velocity at the inlet is negligible compared to the velocity at the valve or device (since it is a much narrower zone). This results into:

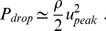
(8)From CFD results, one can also make use of the velocity field in order to estimate a pressure value, emulating in this way what a physician would would measure (fluid speed from Doppler data).

#### Shear stress

The shear stress magnitude, 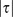, measured in units of 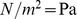 in SI, is defined as.
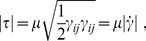
(9)where 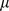 is the dynamic viscosity, 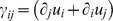 is the shear rate tensor and 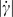 is the effective shear rate that appears in non-Newtonian viscosity models.

Many studies [23]–[25] highlight the key importance of WSS, the shear stress 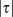 at vascular walls, as an indicator of atherosclerosis and risk of aneurysm development, besides its relevance in terms of biological processes in blood and arterial walls.

#### Hemolysis

The normalized index of hemolysis (NIH) is a measure of the amount of blood damage caused by shear stresses found in a given flow domain. The NIH is in units of 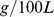 and can be computed as.
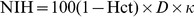
(10)



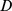
 stands for ‘blood damage’ and it actually represents the change of hemoglobin content of blood (in the literature, sometimes it is represented by 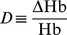), 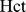 is the hematocrit count (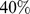 for this study) and 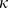 is the hemoglobin content of blood (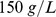 for this study). We discuss in the next section how to compute the blood damage prediction from a CFD physiological reconstruction.

### Differential Equation Describing the Change in Hemoglobin Content

The change in hemoglobin content, 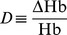, is computed from physiological reconstruction. There are several ways of doing so (see refs. therein [26]), although it is quite extended to use [27]’s integral expression as starting point in order to derive differential expressions,
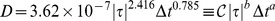
(11)being 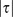 the shear stress (see Eq. (9)) over a particle (in a Lagrangian way) and 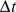 the exposure time.

Following [28]’s notation, in this section we shall work with the generic 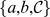 coefficients, also including a wider family of integral expressions of the form of Eq. (11). It is clear that 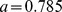, 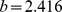 and 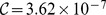 in Giersiepen’s model. Giersiepen’s et al.’s integral expression was derived experimentally under conditions of constant shear stress. The fact that the coefficient 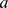 is smaller than unity indicates that damage growth is sublinear in time –a double exposure time translates into less than factor 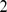 in the growth of blood damage.

We are interested in computing blood damage in any physiological situation, and then shear stress is neither uniform nor constant. In order to do so, a differential form of Eq. (11) must be obtained. This is, we are interested in deriving an equation of the form.
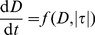
(12)such that if 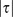 is constant, Eq. (11) is recovered. Notice that the generic function 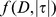 might also explicitly depend on time, 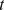, –this is, 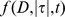. This approach was adopted, for instance, by ref. [29]. In section Results and Discussion we compute and discuss the numerical differences obtained by using this approach.

In order to obtain a differential equation like Eq. (12), Giersiepen’s Eq. (11) can be first linearized in time and then differentiated,
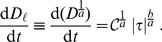
(13)


This approach was first presented by [30] and usually 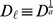 is defined as a new quantity, so-called *linear damage* since it grows linearly with time. Then, the differential equation for actual blood damage, 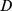, can be straighforwardly obtained,
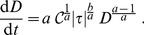
(14)


The consequences of the sublinear dependency (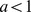) of blood damage with time can be observed in this differential equation: the exponent of the damage factor appearing at the right-hand-side in Eq. (14) will be negative, which means that the damage growth rate decreases the more damaged blood cells are.

Despite Eq. (14) formally being the differential equation for blood damage, the standard procedure when numerically solving a differential equation with a negative exponent on the field that is being integrated, like in our case, is to first perform a change of variables that would return us to linear damage differential equation (13). In any case, it is important to remark here that the linear damage 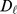 is just a mathematical auxiliary variable, useful to integrate along pathlines, but lacking any physical meaning. In particular, if one wants to compute average damage values, such average must be performed over the actual blood damage, 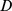, rather than over the linear one. In Appendix S2 we show analytically that this potential misunderstanding would lead to blood damage overestimations of more than 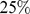.

#### Including linear damage as an additional evolution field of the PDEs

One can replace the Lagrangian time derivative in (13) by its Eulerian form plus an advection term, obtaining a PDE for the linear damage, namely.
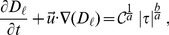
(15)where 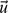 is the fluid velocity and 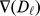 is the linear damage gradient.

This allows us to include now the linear damage 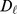 as an additional dynamical field that can be evolved together with the density and linear momentum, governed by the Navier-Stokes equations. This equation can also be combined with mass conservation equation in order to have it rewritten in a conservative form,
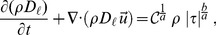
(16)where 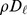 will be the conservative field. The full evolution system is given then by this PDE, plus Eqs. (1)-(2). In section Results and Discussion, we present and discuss the numerical results.

#### Blood damage analytical result for axisymmetric flows

In Appendix S2 we derive an analytical approximation of the averaged damaged hemoglobin count for a viscous Newtonian fluid through a known axisymmetric geometry defined by a single function of the radius along the main axis, 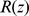. Allowing for [27]’s coefficients, it results into:
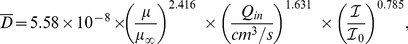
(17)

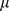
 being the Newtonian blood viscosity, the constant 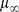 set to 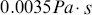, 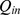 the input volumetric flow rate and 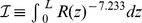 given by the channel shape assuming 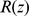, 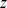 and 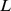 are in 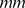, i.e. 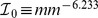. This expression can provide a first approximation (a lower limit, as we shall see in section Results and Discussion) of the damaged hemoglobin count of any axisymmetric geometry, only requiring to compute the integral 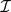, which only depends on the channel geometry and in most cases can be performed analytically.

## Results and Discussion

In this section we aim to discuss the significance and impact of non-Newtonian blood effects on various relevant physiological parameters reconstructed from CFD simulations, and also to validate our DNS code against FDA’s experimental results from their interlaboratory study [1].

### Conservation of Mass Errors

**Figure 3:**
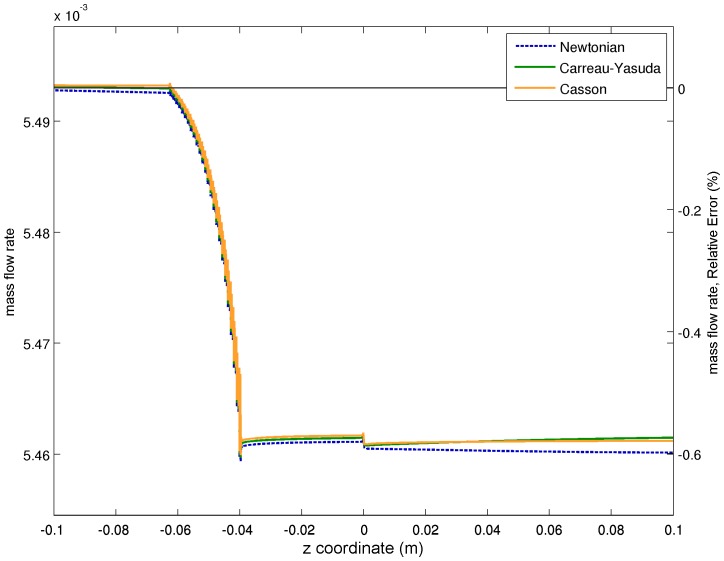
Relative error on mass flow rates, 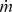, obtained by integrating the axial velocity profiles (multiplied by the density) at different positions along the axis.

Mass conservation is a fundamental law explicitly included in the Navier-Stokes equations (see Eq. (1)). Any deviation of the CFD predicted mass flow rate from the theoretical inlet, 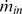 in Eq. (7), represents a numerical error. For this reason, it is quite important to monitor the mass flow rate along the nozzle’s geometry as a key validation indicator of the simulation results. Figure 3 plots the relative error of the CFD predicted mass flow rates versus 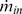, as a function of the axial position. We can observe that mass flow rate remains almost constant for 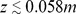 and for 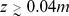, and there is a mass flow rate loss of ∼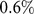 in between these two positions, which corresponds exactly with the conical collector part of the nozzle’s geometry (see Figure 1).

The reason why most of the conservation of mass errors concentrate in the conical collector is because it is there where the nozzle’s shape is harder to fit in a regular orthogonal mesh. Indeed, we have observed in some other preliminary results that these errors can be reduced by more than an order of magnitude if one allows for subcell resolution by working with fractional volume-of-fluid (VOF) methods [31]–[33] at each cell. Notice that mass conservation errors are almost independent of the blood viscosity model.

It is also worth pointing out that these flow rate errors are just about a half percent in our case and converge as the mesh is refined, as discussed in Appendix S1.

### Axial Velocity Along Centerline and Radial Cuts Profiles

**Figure 4:**
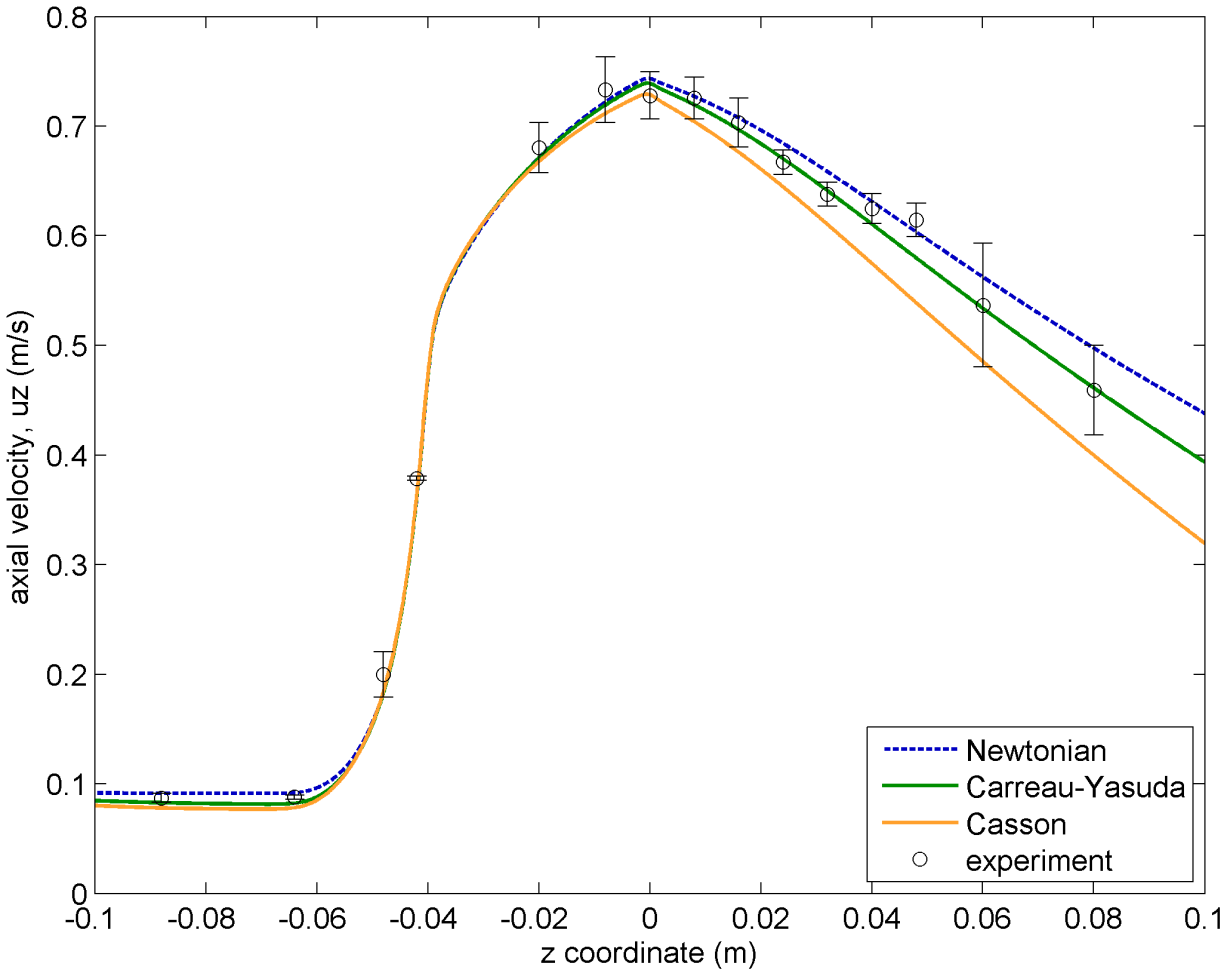
Axial velocity along the nozzle centerline for the three viscosity models. CFD results (lines) are compared to experimental data represented by their means and 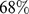 confidence intervals (i.e.: 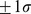).

The axial velocity profile along the centerline is a key indicator to validate the performance of CFD predictions, on the one hand because the plot includes information from all the different nozzle regions, and also because the experimental velocity values were precisely measured using PIV techniques. Figure 4 plots CFD predictions of the axial velocity profiles along the centerline for the three different viscosity models together with the experimental means and their 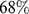 confidence intervals. Non-Newtonian effects on the axial velocity are small in the region upstream the sudden expansion, but they become more significant downstream that point, in the recirculation zone. The Newtonian model underestimates blood viscosity at low shear stress rates, which is the reason why Newtonian axial velocity is higher than the one obtained with the other models. The same thing happens when comparing the CY model with respect to Casson’s. The latter tends to overestimate the viscosity values at very low shear stress rates (see Figure 2), and one gets lower axial velocity values as a result [34], [35].

By comparing Newtonian versus CY results, we can see that non-Newtonian effects induce differences on the axial velocity which grow up to 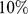, being larger in any case than experimental confidence intervals. Some authors consider than these moderate differences would not suggest the need of including non-Newtonian effects to obtain reliable CFD predictions, even for these low Reynolds numbers [3], [4]. We will rather take this as an indication of a significant source of errors, which could have consequences when looking at some physiologically relevant parameters, as we will see below.

**Figure 5:**
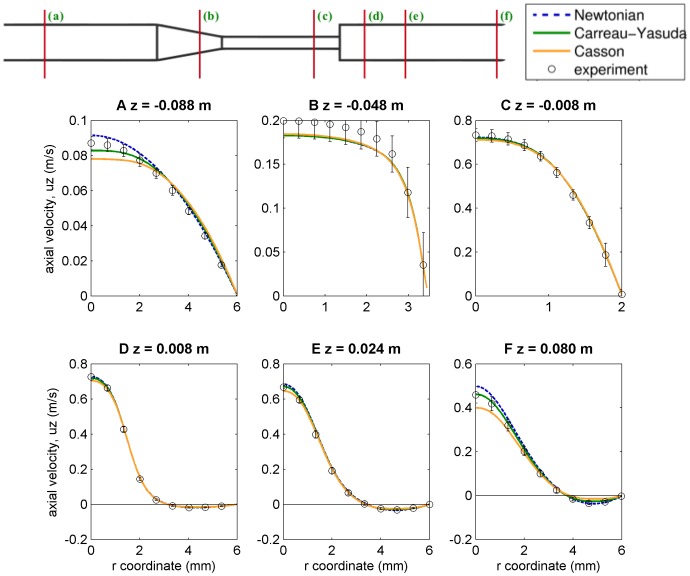
Axial velocity profiles (CFD results and experimental values) along radial cuts at different positions of the nozzle.

The study of the axial velocity can be completed by plotting this field along radial cuts at different positions of the nozzle (see Figure 5). Again, CFD predictions agree very well with experimental results, finding just slight discrepancies in the conical region. The differences between different viscosity models do not exceed 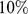. The three subplots at the bottom correspond to radial cuts downstream of the sudden expansion, where some fluid recirculation (negative axial velocities) can be observed.

### Pressure Drop

Another direct measure obtained in the *in vitro* experiments run by the laboratories participating in the FDA’s challenge was pressure along the centerline. This is indeed a very relevant quantity from the medical point of view, as it is often used to detect, for instance, aortic valve stenosis. Clinically, with actual patients, the pressure drop along a valve or device cannot be directly measured. Instead, physicians make use of the simplified Bernoulli equation to non-invasively estimate the pressure drop at the narrowing from velocity measures obtained using Doppler techniques (see Eq. (8)).

Besides the experimental *in vitro* measures, we have discussed several other ways to obtain pressure drop values. From CFD simulations, pressure can be obtained as an independent field if one is using a slightly compressible model, like in this article.

**Figure 6:**
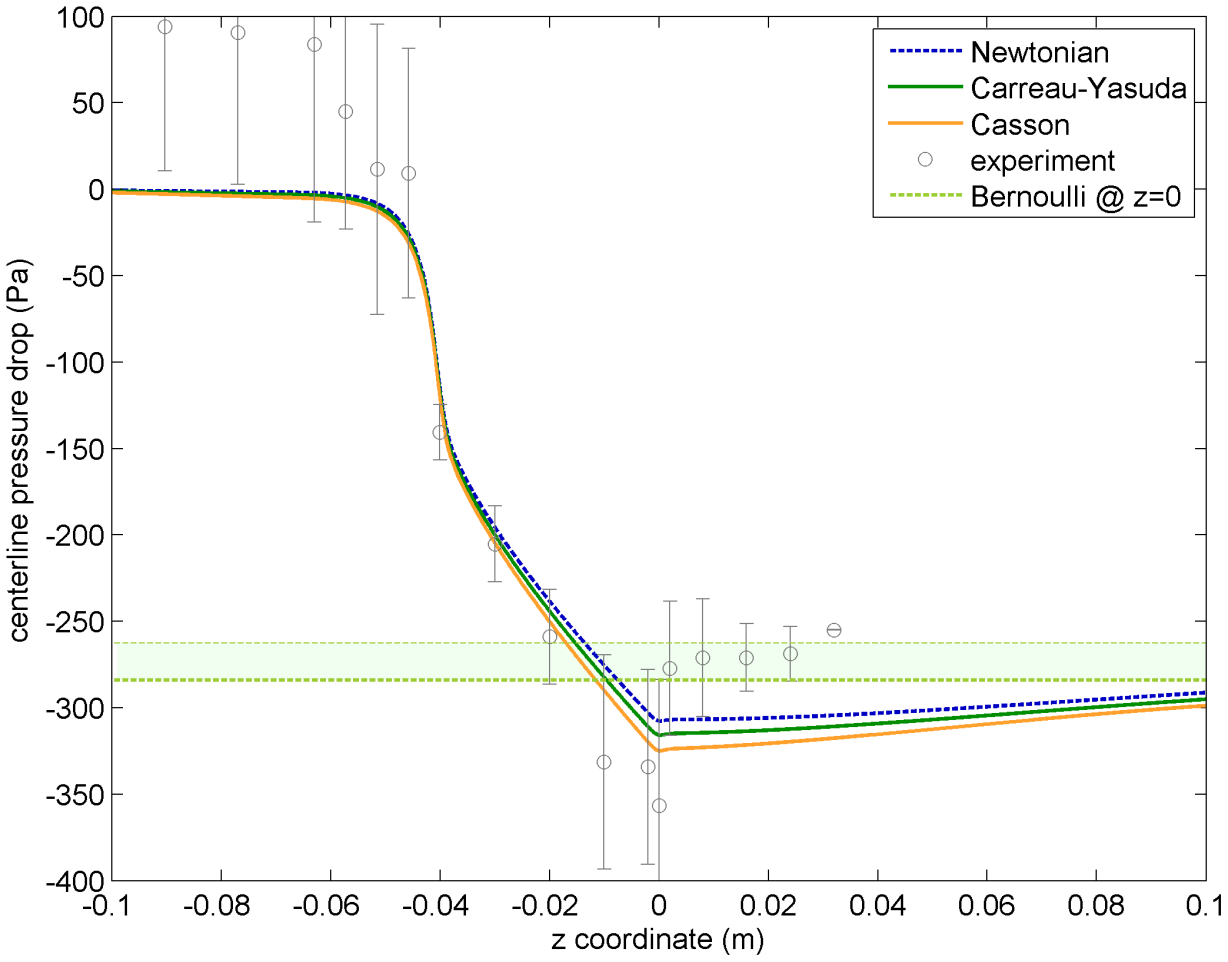
CFD predicted pressure drop values along the nozzle centerline for the three different viscosity models (lines), together with maximum pressure drop value derived from peak velocity value using Bernoulli’s equation (thick dashed horizontal line). Experimental data are represented in light gray color because of their lack of reliability due to experimental errors, as pointed out in FDA’s report [1].

Figure 6 plots the pressure drop (with respect to the pressure value at the inlet) predicted by the CFD simulations using the three different viscosity models, plus the value obtained from the simplified Bernoulli equation using peak velocity values, plus the experimental means with confidence intervals. Notice that the experimental data suggest a faster recovery of the post-stenotic pressure, which is not replicated by any of the three models. This difference can be put in context by noticing that, according to FDA’s report [1], experimental values of pressure for this particular Reynolds number (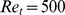) were not reliable (experimental points are plotted in light gray for this reason). Moreover, our curves are much closer to the experimental points than the CFD results presented in the FDA report (see Figure 4.a in [1] for details).

The predicted pressure drop at the sudden expansion is 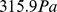 for the CY viscosity model; 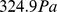 for the Casson model (a 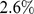 overestimation w.r.t. CY); 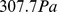 for the Newtonian model (a 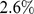 underestimation w.r.t. CY) and 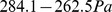 for the simplified Bernoulli equation (a 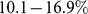 underestimation w.r.t. CY). In light of these results, for this particular geometry and flow regime, non-Newtonian effects have a limited effect concerning CFD predicted pressure values. The non-invasive method to estimate the pressure drop using the simplified Bernoulli equation has instead more than a 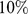 underestimation (see ref. [21] for a discussion of the relevance of this error). Notice that an underestimation of the pressure drop means that a potential stenosis would be underestimated as well.

### Shear Stress Profiles and WSS

**Figure 7:**
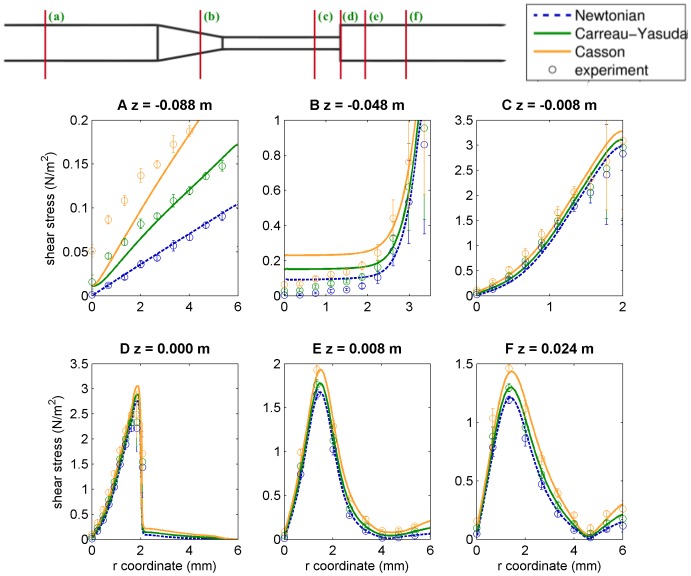
Blood shear stress profiles along radial cuts at different positions along the nozzle’s axis. Lines represent CFD predictions and indirect experimental data is represented by their means and confidence intervals. Since shear stress values obtained from experimental data actually require to set a viscosity model, here we plot three different experimental data sets (Newtonian, CY and Casson), one for each viscosity model.

**Figure 8:**
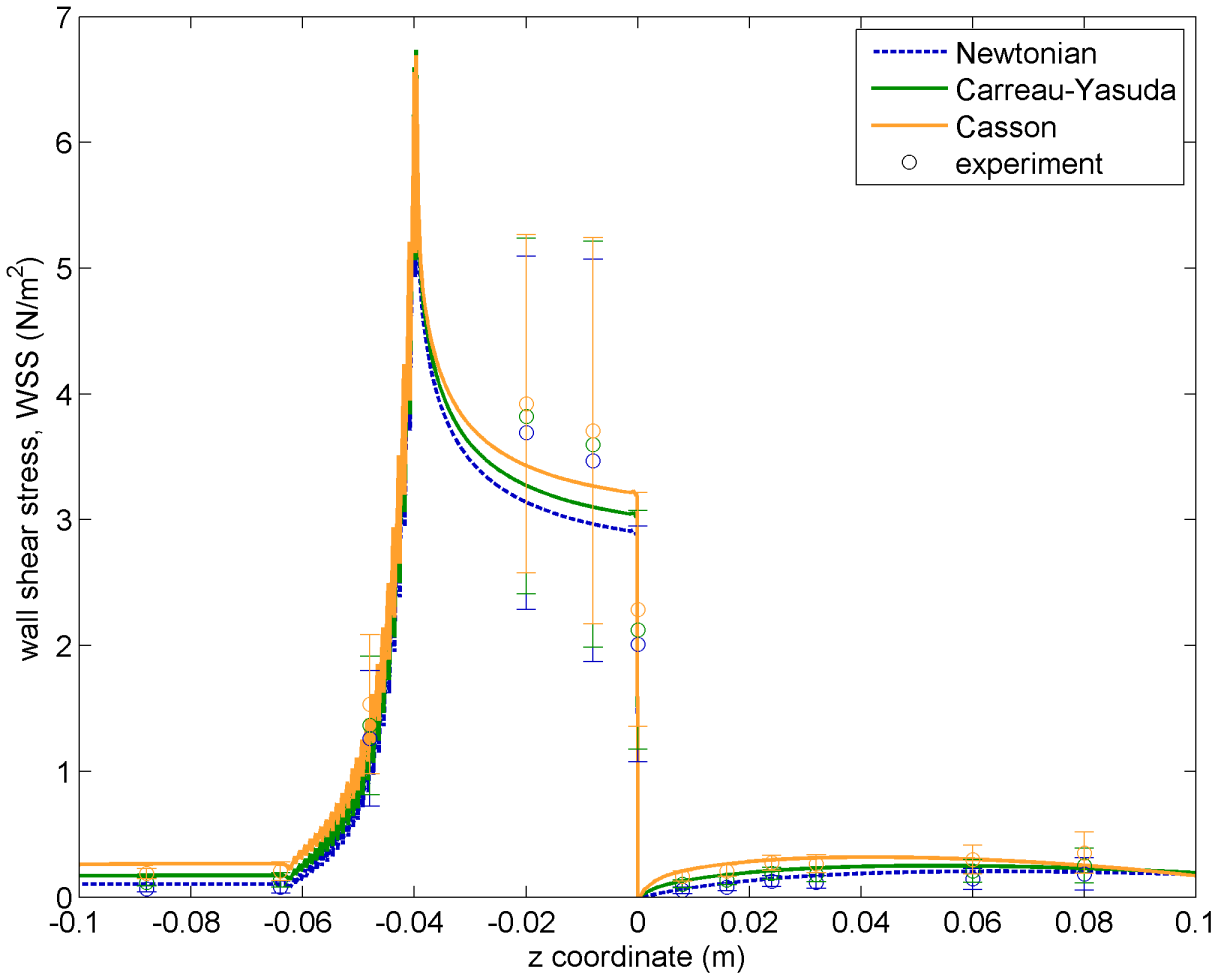
WSS values as a function of the axial position. Lines represent CFD predictions for the three viscosity models considered (using the same color code as in previous figures), whereas experimental results (indirectly measured from velocity profiles and assuming each of the three viscosity models studied in this article) are plotted by their mean values and 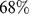 confidence intervals.

It is perhaps in the estimation of shear stress values where a CFD contribution can be more relevant to the medical and bioengineering community. This is because, on one hand, experimental shear stress rates are difficult to obtain. Indeed, shear rates in the FDA’s interlaboratory experiments were calculated indirectly from the velocity field (computing numerical derivatives from experimental points) [1] and then converted to shear stress values assuming a specific viscosity model (results depend on the chosen model, see the discussion below). As matter of fact, experimental errors raise in regions near the wall due to difficulties in imaging velocities there (see anticipating results in Figs. 7 and 8). On the other hand, shear stress values provide very valuable information, which may be used to estimate arterial wall or valve mechanic stresses, blood damage indicators, calcification rates, etc.

Figure 7 plots blood shear stress profiles along radial cuts at different positions along the nozzle from CFD predictions using the three different viscosity models, and also the experimental values, obtained in each case by assuming the corresponding viscosity model. We can see looking at sub-panels (b), (c), and (d), that shear stress experimental errors substantially increase near the wall, which turns out to be the region where more critical the data is regarding estimates of WSS and also where the most important contributions to the blood damage integral occur. Upstream of the sudden expansion (sub-panels (a) to (c)), the maximum shear stress is found near the wall, whereas downstream of that point (sub-panels (d) to (f)), a recirculation zone is created and is, indeed, at the transition zone between forward and reverse flow where the maximum shear stress values are recorded.

Comparing shear stress profiles between different viscosity models, we can see that more important relative differences seem to be upstream of the conical region (sub-panels (a) and (b) of Figure 7), where the slowest and most laminar flow regime occurs. However, one should notice the different scales in the shear stress axes to realize that these differences correspond to very low shear values. Bigger contributions come from the narrow tube and downstream of the sudden expansion (sub-panels (c) to (f)), where relative differences are more moderate (∼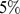) and consistent with typical values found in the literature [3], [4], [36].

Figure 8 plots the WSS values along the proximity of the nozzle’s wall, by calculating the index position of the grid cell the wall and using the WSS of two grid cells below, in order to diminish the staircase effect of inclined geometry without affecting the results. In this figure, one can appreciate even better how the big experimental errors are recorded in the most crucial regions: from the conical taper (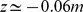) to the sudden expansion (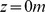), where most of the blood damage is being produced. The maximum WSS is produced at the transition point between the conical and narrow regions. It is a point, together with the sudden expansion, that caused trouble to some of the CFD submissions at the FDA’s challenge (see Figure 13 of [1]) since it requires a fine definition of the wall and of its interaction with blood. As it was written in the previous paragraph, relative differences between the three viscosity models are larger at low WSS values (e.g. upstream the conical region, 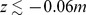). However the relevant contributions come from the narrower regions of the nozzle, where relative differences are ∼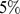 between models; in particular, at a point just upstream the sudden expansion (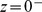) measured CY’s WSS values are 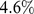 larger than Newtonian and 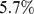 smaller than Casson.

### Hemolysis

#### Analytical approximation

We have proposed an analytical approximated expression to compute the averaged damaged hemoglobin for axisymmetric flows, Eq. (17). The full derivation is provided in Appendix S2, where we assume a Poiseuille flow at each ‘z’ with constant volumetric flow rate, 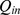. This may be in some cases a rough approximation, but the benefit is that the final expression only depends on the geometry 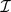, the fluid viscosity 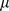, and the volumetric inflow rate 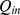. For the particular problem studied in this article, the Newtonian blood viscosity is 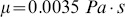 and the input volumetric flow rate for the 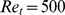 problem, is 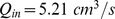. The integral 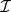 can be computed from the geometrical information prescribed by the FDA, which can be mathematically expressed as a piecewise function (all distances, 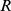 and 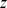, are in 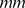):
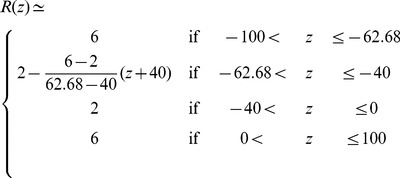
(18)One gets then from the definition:
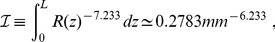
(19)which leads to a damaged hemoglobin count 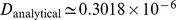, after applying Eq. (17). Finally, the analytical lower limit of the averaged NIH can be obtained from Eq. (10),
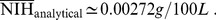
(20)


#### CFD results and impact of non-Newtonian effects

The analytical NIH result, Eq. (20), provides a first guess on the magnitude of hemolysis rates. It is a calculation that does not require any dynamical information from CFD simulation, only the geometrical shape and the input flow rate. The purpose of this subsection is to study the corrections to this guess introduced by full CFD simulations, using either the Newtonian blood model or the two non-Newtonian models described in section Materials and Methods.

NIH results can be obtained from CFD simulations by using two different approaches:

- *First approach:* one can take the velocity and shear stress magnitude fields of the steady solution (the latter can be computed from spatial derivatives of the former, Eq. (9)) and integrate the ordinary differential equation for the linear damage, Eq. (13), along several pathlines (or streamlines, as they are the same under steady conditions) distributed over a radial section of the nozzle. Each pathline will accumulate different linear damage values, which have to be converted to actual blood damage hemoglobin counts, 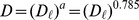. Then *and only then*, the different blood damage values are averaged, weighted with the flow rate at the seed point of each pathline (i.e. using the same idea as in Appendix S2), and the averaged NIH is finally computed. The process of this first approach can be summarized as follows:
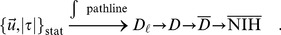
(21)
- *Second approach:* one could rather adopt the position suggested by [30] of including linear damage, 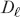, as an additional evolution field of the PDEs system. This linear damage field can be converted to actual blood damage, 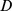, and then to NIH, which therefore can be represented over the entire domain, as any other field, displaying the accumulated blood damage of the particles at every point.

Both approaches are expected to be equivalent if we look at averaged values, although they are quite different from the data analysis point of view: the first one can be seen as a post-processing analysis using the CFD predicted velocity field, whereas in the second one the blood damage count is already computed within the PDE evolution system, giving more detailed information on the damage distribution.

Following the first approach, the averaged NIH values that are obtained for the three different viscosity models are:
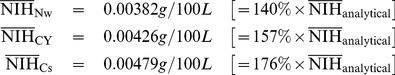
(22)


**Figure 9:**
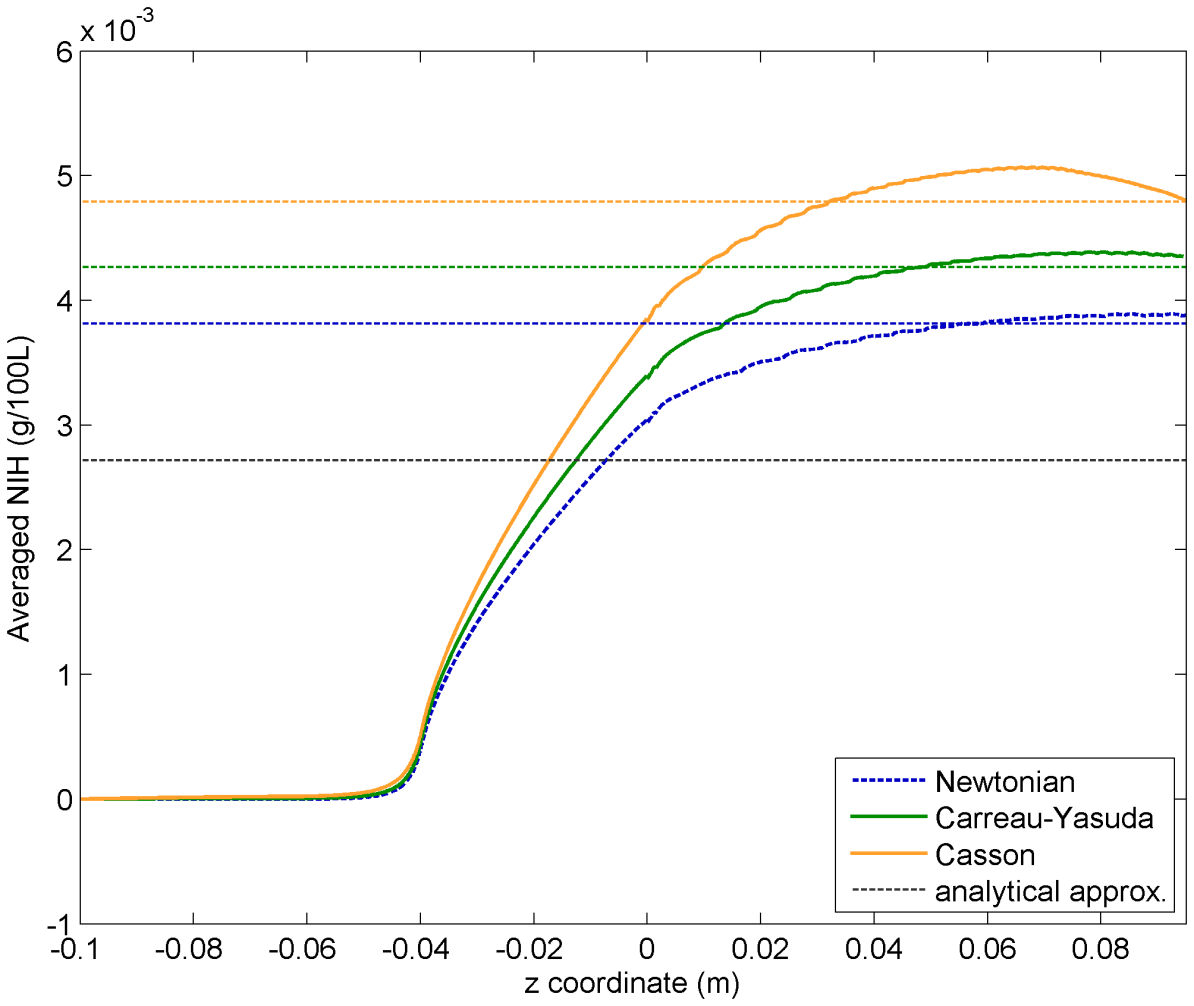
Flux-weighted NIH average values (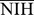) using the different viscosity models, together with the analytical lower limit. Thick curved lines correspond to the values obtained from computing the flow-weighted averages over radial cuts along the main axis using the NIH evolution field included as a PDE. Thin dashed lines represent the averaged 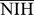 values at outflow given in Eqs. (22), obtained from a post-processing analysis of the velocity field.

In order to make the NIH field values obtained from the second approach comparable to these averaged results, one can compute flow-weighted averages along radial cuts (see Appendix S2), obtaining 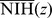 curves. Figure 9 plots these averaged NIH curves obtained from the lineal blood damage count as an extra field of the evolution PDEs (i.e. the second approach) together with the reported [see Eqs. (22)] values from the first approach. Both approaches lead to very similar results, proving that they are actually equivalent. The analytical lower limit obtained in Eq. (20) is also plotted in the same figure.

By comparing hemolysis results for the different viscosity models, we see the same qualitative behaviour as in shear stress curves. A qualitative conclusion is that Newtonian models tend to underestimate blood damage counts.

In regard to the discussion about the relevance of non-Newtonian effects, one could argue that 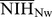 is only 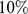 smaller than its non-Newtonian (CY) counterpart, an error which could be dismissed (or not, because it is an underestimation). However, this conclusion is misleading, since it amounts to assume ‘zero-knowledge’ about the hemolysis rates in our study, that is 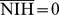, instead of using the analytical lower limit. From this perspective, it turns out that performing a full CFD simulation corrects the analytical first guess 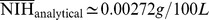 to the Newtonian value 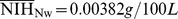, this is, a 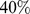 correction. Actually, a CFD simulation not only provides a more realistic NIH value, but also full 4D reconstruction of relevant physiological fields, so it is of course always worth to perform it. Then, including non-Newtonian effects (which, at least in our implementation, has a negligible impact on the simulation code performance) adds up another 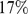 correction, which represents almost half of the improvement obtained from 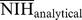 to 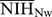. Schematically this statement can be represented as follows,
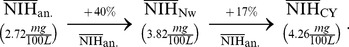
(23)


NIH is the standard clinical index used to report hemolysis. Also, one of the most interesting potential applications of CFD simulations in medical devices is the possibility of being able to quickly evaluate the effect of design changes on device performance by resolving localized (in time and space) blood damage effects [29]. In this context, a 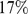 difference in NIH predictions due to the choice of a simplified blood rheological model may be unacceptable, perhaps making the difference between turning down a valid design or accepting a too hemolytic one. In this sense, our results suggest that non-Newtonian viscosity models can be relevant when trying to obtain reliable hemolysis results from CFD simulations.

#### Results using other methods found in the literature

[29] proposes a different way of computing the change of hemoglobin content, 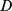. Also starting from [27] integral expression, Eq. (11), they derive a differential equation where 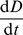 explicitly depends on time, 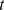. In particular,
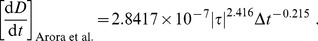
(24)


Notice that this means that the growth rate of blood damage depends on the time origin choice, no matter what damage and shear stresses blood cells underwent in the past. Once a time origin is set, it can be applied to the results of the problem (in the steady regime in order to obtain a numerical result for the average NIH which can be compared with CY’s Eq. (22): 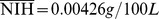 [here CY model is also being used],
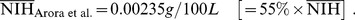
(25)


The value obtained with this method is almost a factor 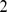 smaller. This is if the time origin is set at the moment when fluid particles passed through 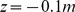. If the initial time was set before, the resulting value would be even smaller, as the relevant contributions to blood damage would occur at larger 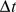.

At the end of Appendix S2 we discuss the effects of not transforming back the auxiliary linear blood damage count, 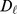, to actual blood damage, 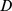, just after the integration along pathlines and always *before* doing any other calculation such as averaging. This would mean, instead of proceeding according to the process pictured in (21), to proceed as follows,
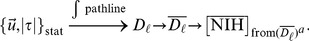
(26)


Using CFD predicted velocity fields for the CY model, one can compute averaged NIH value following (26), and obtain.
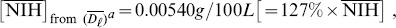
(27)which indeed represents a 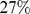 overestimation, as predicted for the blood damage (see Appendix S2), since there is a lineal relation between 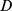 and NIH.

## Conclusions

We have performed CFD simulations of blood flowing through an idealized cardiovascular medical device (see Figure 1) for a throat Reynolds number of 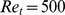, and compared against a recent validation study performed by the FDA [1] based on experimental data from three independent laboratories and some CFD submissions from different research groups. Our simulation code was automatically generated by Simflowny [2], a general-purpose platform for the management of physical models and simulation problems developed by ourselves at the 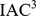. The physiological models we used correspond to the Navier-Stokes equations (see Eqs. (1)-(2)) plus a slightly compressible Equation of State, Eq. (3), and the possibility of using different non-Newtonian viscosity models (indeed, we are following the so-called DNS approach). In particular, we have considered three rheological models for the blood viscosity: *Newtonian*, *Carreau-Yasuda* and *Casson*. Also, we have discussed different hemolysis indicators, deriving an analytical expression that may be used to obtain lower limits on the blood damage (see Eq. (17)) and implementing the most realistic indicators within our CFD environment.

We have validated our results against the experimental data provided by the FDA, obtaining very good agreement. In particular, such validation has been done (a) against theoretical results, such as the conservation of mass; (b) against direct experimental results, such as velocity profiles and pressure drop values; and (c) against indirect measures, such as blood damage and WSS. This latter comparison is harder due to experimental measurement difficulties, specially at the throat, making a point of where CFD predictions can help in physiological and hemolysis studies.

The observed differences between Newtonian and non-Newtonian results in velocity, pressure and shear stress fields can reach up to a 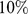, which is consistent with other results found in the literature in more realistic geometries [3], [4]. Actually, these moderate differences led the authors of [3] to suggest that there is no need of including non-Newtonian effects to obtain reliable CFD predictions. However, our conclusions suggest some relevance when looking at the hemolysis results (see paragraphs below). In any case, let us point out that our CFD code can provide this 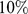 improvement with no significant computational cost.

Also, we have discussed different ways of computing the change of hemoglobin content from CFD, a key ingredient to finally obtain hemolysis indicators, such as NIH. In particular, with steady flows we have (a) taken the steady solution and integrate (14) along pathlines and (b) included the linear damage as an additional evolution field (15) (i.e. defined at each space and time points) and solved the PDE. We have tested both approaches on the FDA’s problem finding consistency between them. We have also explored a third option, consisting in an approximation valid for any axisymmetric flow that allowed us to obtain an analytical expression for the average blood damage only as a function of the geometry (eq. (17)). We also discuss the importance of converting the linear damage 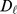 into the actual blood damage 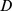 *before* averaging. Otherwise one can get large errors in the average NIH value.

When comparing hemolysis results between Newtonian and non-Newtonian models, we observed in the Newtonian model a 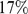 underestimation of NIH (taking the analytical lower limit 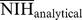 as a common basis). This represents an adjustment of almost half the correction that separates an analytical result from the Newtonian CFD prediction (i.e. the difference that justifies, inter alia, performing a full CFD simulation, see (23)). Our results indicate that non-Newtonian effects are actually amplified in blood damage indices.

The work presented in this article represents the first step towards a more general goal of simulating implanted in-body medical devices using patient-specific data acquired with CT scans or MRI techniques in order to obtain morphology and blood fluxes. Our implementation differs from others found in the literature in different ways (*slightly compressible blood*, *inclusion of non-Newtonian effects*, *non-reflecting characteristic boundary conditions*…); hence, following FDA’s recommendation [1], we have started validating our CFD code against known experimental results finding successful agreements. We have also discussed and successfully tested some hemolysis indicators that shall be used in our future studies. The next steps towards this direction consists in allowing the walls to move and start looking at the performance of our DNS implementation at higher Reynolds numbers, keeping AMR and LES techniques as possible improvements, to be added when required.

## Supporting Information

Figure S1
**Convergence of the Mass flow rate using three resolutions.** We plot the mass flow rate for grids with resolution ratios 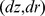,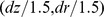 and 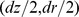. They correspond to the following 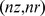 numbers of points: 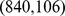,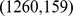 and 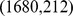. The solutions converge with an 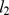 norm of 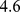. The left axis shows the value of the mass flow and the right axis shows the relative error with respect to the theoretical value.(TIFF)

Figure S2
**Convergence of the Wall Shear Stress using three resolutions.** We plot the WSS for grids with the same resolution ratios as Figure S1. The solutions converge with an 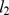 norm of 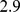 and a 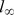 norm of 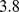.(TIFF)

Figure S3
**Convergence of the normalized index of hemolysis using three resolutions.** We plot the NIH for grids with the same resolution ratios as Figure S1. The solutions converge with an 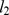 norm of 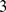.(TIFF)

Figure S4
**Contour lines of blood damage overestimation in the axisymmetric–Poiseuille case if one computes the average on the linear damage,** 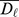 **, instead of first unlinearizying it.** The two axis correspond to the hemolysis coefficients 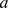 and 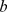, the dashed lines representing Giersiepen et al. values. The plot has been restricted to the sublinear regime, *a*<1.(TIFF)

Appendix S1 (PDF)

Appendix S2 (PDF)
