# Supplementary figures and images for: FDA’s Nozzle Numerical Simulation Challenge: Non-Newtonian Fluid Effects and Blood Damage
Source: PLoS One. 2014 Mar 25;9(3):e92638. doi: 10.1371/journal.pone.0092638 (PMC3965442; doi:10.1371/journal.pone.0092638)

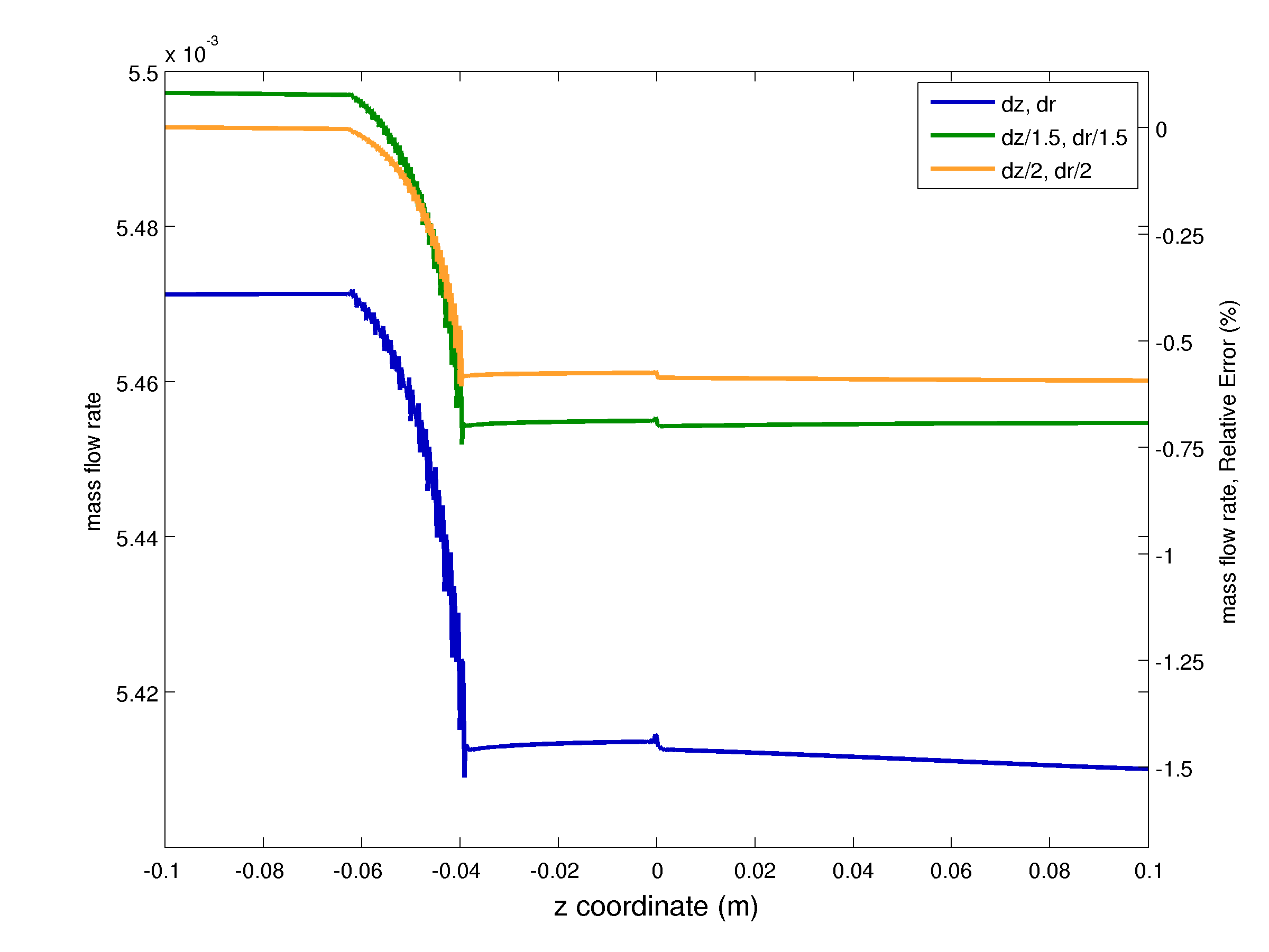

Supplement: Figure S1 — Convergence of the Mass flow rate using three resolutions. We plot the mass flow rate for grids with resolution ratios , and . They correspond to the following numbers of points: , and . The solutions converge with an norm of . The left axis shows the value of the mass flow and the right axis shows the relative error with respect to the theoretical value. (TIFF) [file pone.0092638.s001.tiff]

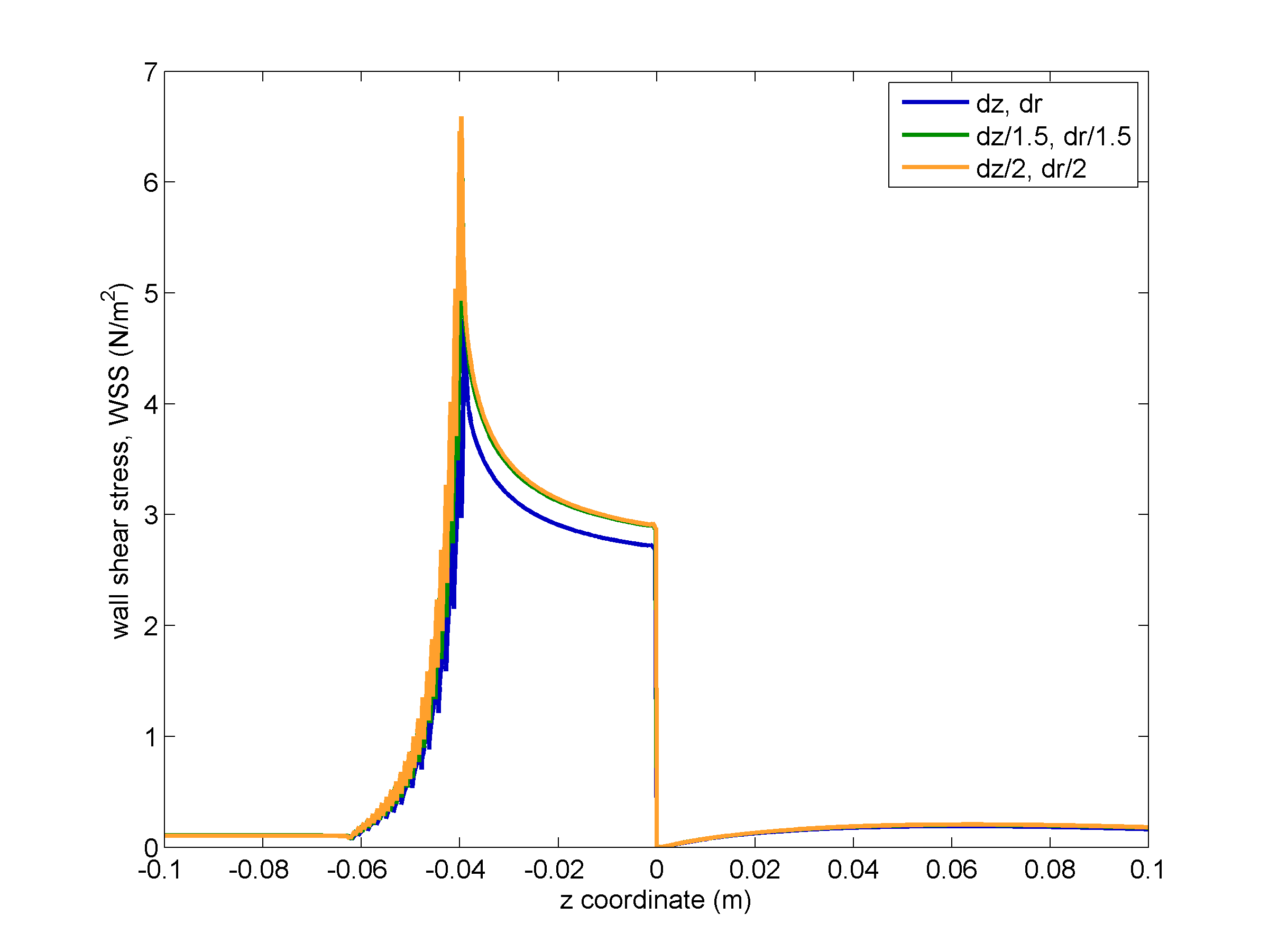

Supplement: Figure S2 — Convergence of the Wall Shear Stress using three resolutions. We plot the WSS for grids with the same resolution ratios as Figure S1. The solutions converge with an norm of and a norm of . (TIFF) [file pone.0092638.s002.tiff]

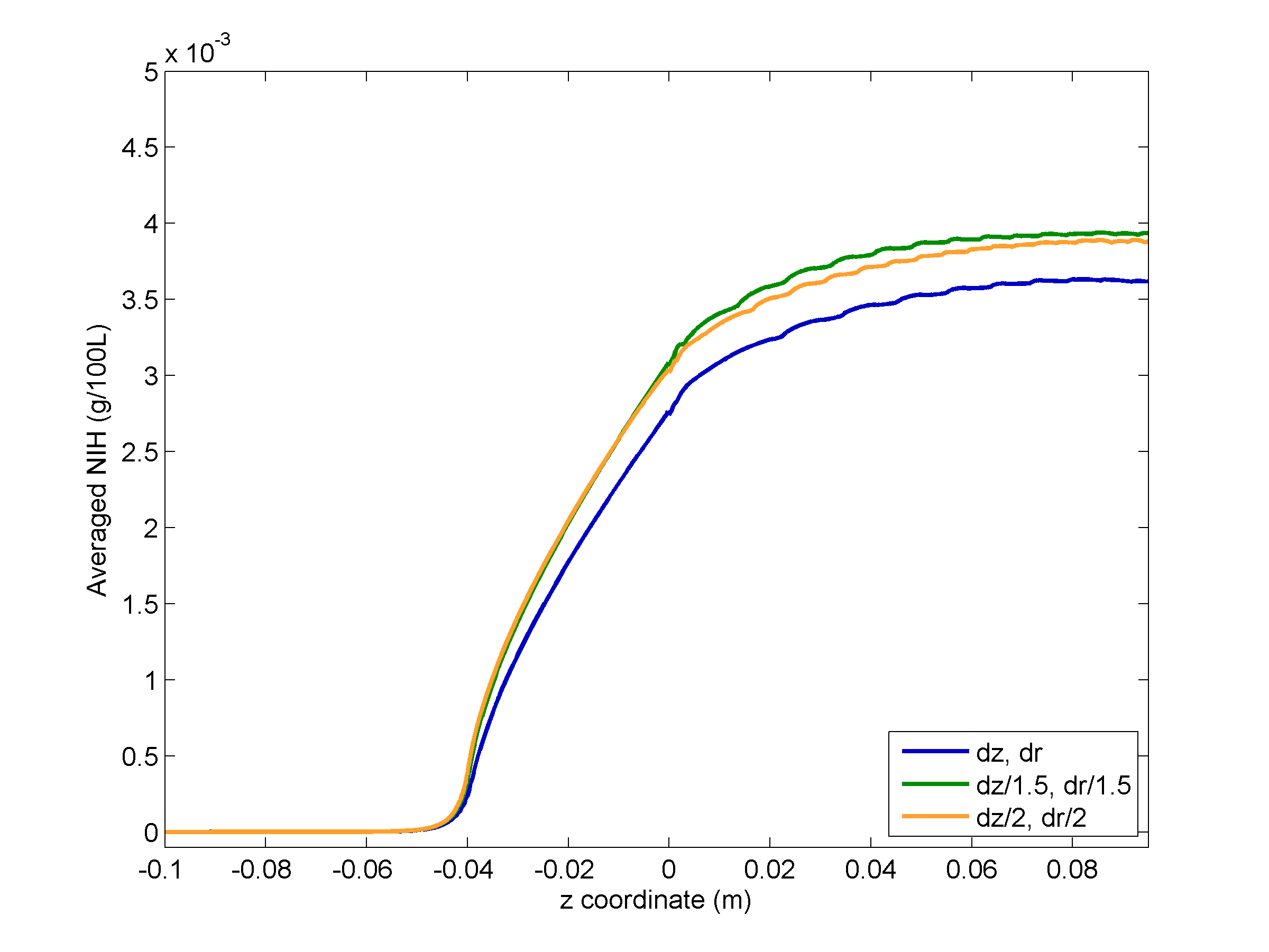

Supplement: Figure S3 — Convergence of the normalized index of hemolysis using three resolutions. We plot the NIH for grids with the same resolution ratios as Figure S1. The solutions converge with an norm of . (TIFF) [file pone.0092638.s003.tiff]

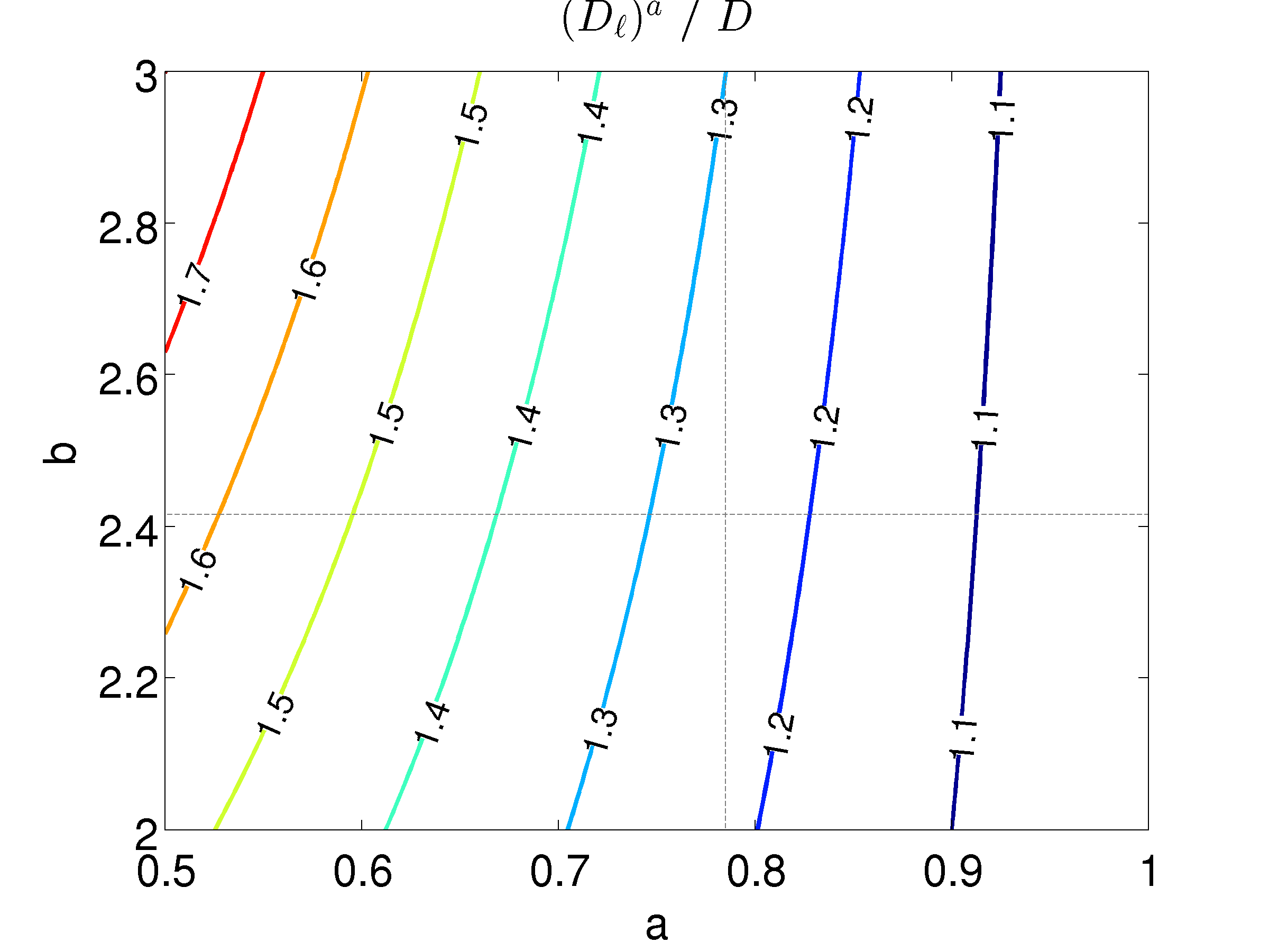

Supplement: Figure S4 — Contour lines of blood damage overestimation in the axisymmetric–Poiseuille case if one computes the average on the linear damage, , instead of first unlinearizying it. The two axis correspond to the hemolysis coefficients and , the dashed lines representing Giersiepen et al. values. The plot has been restricted to the sublinear regime, a<1. (TIFF) [file pone.0092638.s004.tiff]
