# Supplementary material for: FDA’s Nozzle Numerical Simulation Challenge: Non-Newtonian Fluid Effects and Blood Damage
Source: PLoS One. 2014 Mar 25;9(3):e92638. doi: 10.1371/journal.pone.0092638 (PMC3965442; doi:10.1371/journal.pone.0092638)
Supplement: Appendix S1 — (PDF) [file pone.0092638.s005.pdf]

# Appendix S1: Convergence tests

Following Roache [1], we use several simulations with different resolutions to check the convergence rate of our simulations. As a good error indicator, we first select the mass flow rate, which should be constant along the nozzle due to the continuity equation. In Figure S1 we plot the mass flow for three different resolutions, refined in both directions. The mass shows convergence with an $l_2$ norm of 4.6, which, allowing for the mixture of spatio-temporal orders of the numerical algorithm, is an excellent result. Nevertheless, the convergence to a non-constant profile shows that the mass flow does not approach a constant rate, as expected. Instead, a systematic error is introduced by the treatment of the Noozle's geometry. In the right axis we show the total relative error of the solutions, which should converge to 0. It is clear that the error appears after the nozzle straitening cone. We are aware that the numerical treatment of the inclined wall produces an artificial staircase effect. The resulting error decreases with space resolution, but with a lower convergence rate. These errors can be reduced by more than an order of magnitude if one allows for subcell resolution by working with fractional volume-of-fluid (VOF) methods [2–4] at each cell.

In any case, it should be noted that the maximum relative error, even with the coarsest grid, is less than 1.5%, whereas calculations of Ref. [5] (Figure 2) have errors of the order of a few percent. Note that the results shown in the paper correspond actually to the highest resolution, where our relative error is less than 0.6%.

Furthermore, given that mass flow might be considered insufficient for demonstrating mesh convergence, we have applied the same procedure to the most important indicators: WSS and NIH. The WSS is a harsh indicator of numerical convergence, as it has blunter profiles and high gradients. In Figure S2 we plot the WSS for the same three different resolutions as before. The $l_2$ norm is 2.9, which is a very good result. The $l_\infty$ norm gives an even better convergence of 3.8, indicating that the spike is well resolved. The lower convergence rate in the $l_2$ norm is due to the extensive boundary effect (the accuracy of the numerical algorithm is lowered to third order near the boundaries). In Figure S3 we plot the NIH, which converges as well with an $l_2$ norm of 3.

# References

1. Roache PJ (1998) Verification of codes and calculations. AIAA Journal 36: 696–702.

2. Hirt C (1981) Volume of fluid (VOF) method for the dynamics of free boundaries. Journal of Computational Physics 225: 201–225.

3. Sussman M, Smereka P (1994) A level set approach for computing solutions to incompressible two-phase flow. Journal of Computational Physics 114: 146–159.

4. Miller GH, Colella P (2002) A conservative three-dimensional Eulerian method for coupled solid-fluid shock capturing. Journal of Computational Physics 183: 26–82.

5. Stewart SFC, Paterson EG, Burgreen GW, Hariharan P, Giarra M, et al. (2012) Assessment of CFD Performance in Simulations of an Idealized Medical Device: Results of FDAs First Computational Interlaboratory Study. Cardiovascular Engineering and Technology 3: 139–160.
