# Supplementary material for: FDA’s Nozzle Numerical Simulation Challenge: Non-Newtonian Fluid Effects and Blood Damage
Source: PLoS One. 2014 Mar 25;9(3):e92638. doi: 10.1371/journal.pone.0092638 (PMC3965442; doi:10.1371/journal.pone.0092638)
Supplement: Appendix S2 — (PDF) [file pone.0092638.s006.pdf]

Appendix S2: Deriving a blood damage analytical result for axisymmetric flows

Let us consider a viscous Newtonian fluid through a known axisymmetric geometry (this is, we are working with cylindrical coordinates $\{r, \phi, z\}$ and neglect any angular dependency) defined by a single function of the radius along the main axis, $R(z)$. As a first approximation, we can consider the flow as a steady Poiseuille flow at each 'z' with constant volumetric flow rate, Q,

$$u_z(r,z) = u_0\left[1-\frac{r^2}{R(z)^2}\right] = \frac{2Q_{in}}{\pi R(z)^2}\left[1-\frac{r^2}{R(z)^2}\right] \tag{1}$$

$$u_r(r,z) = 0\,. \tag{2}$$

From the velocity profile, one can compute the shear stress magnitude, $|\tau|$, Eq. (9) of the main text, resulting into

$$|\tau(r,z)| = \frac{4\mu Q_{in}}{\pi}\frac{r}{R(z)^4}\,. \tag{3}$$

Assuming streamlines (coincident with pathlines in steady flows) to be such that $r' \equiv r/R(z)$ is constant, we can compute the accumulated damaged hemoglobin count along the pathline, first integrating the linear damage

$$\begin{aligned} D_\ell(r') &= \mathcal{C}^{\frac{1}{a}}\int |\tau|^{\frac{b}{a}}\,dt = \mathcal{C}^{\frac{1}{a}}\int_0^L |\tau|^{\frac{b}{a}}\frac{1}{u_z}\,dz \\ &= \mathcal{C}^{\frac{1}{a}}(2\mu)^{\frac{b}{a}}\left(\frac{2Q_{in}}{\pi}\right)^{\frac{b}{a}-1}\frac{r'^{\frac{b}{a}}}{(1-r'^2)}\int_0^L R(z)^{2-\frac{3b}{a}}\,dz \\ &\equiv \mathcal{C}^{\frac{1}{a}}(2\mu)^{\frac{b}{a}}\left(\frac{2Q_{in}}{\pi}\right)^{\frac{b}{a}-1}\frac{r'^{\frac{b}{a}}}{(1-r'^2)}\,\mathcal{I}\,; \end{aligned} \tag{4}$$

and then reverting the linearization

$$D(r') = (D_\ell)^a = \mathcal{C}(2\mu)^b\left(\frac{2Q_{in}}{\pi}\right)^{b-a}\mathcal{I}^a\,\frac{r'^b}{(1-r'^2)^a}\,, \tag{5}$$

which is the final result for the blood damage accumulated from $z = 0$ up to $z = L$ at constant $r' \equiv r/R(z)$.

Actually, we can go one step further, since the result we are really interested in is the flow-weighted average of the damaged hemoglobin count, which can be obtained from the previous result, $D(r')$, weighted with the flow rate at each r',

$$\overline{D} = \frac{\int_0^1 D(r')u_z(r',z=0)\,r'\,dr'}{\int_0^1 u_z(r',z=0)\,r'\,dr'} = 2\mathcal{C}(2\mu)^b\left(\frac{2Q_{in}}{\pi}\right)^{b-a}\mathcal{I}^a\frac{\Gamma(2-a)\Gamma(\frac{b}{2}+1)}{\Gamma(3-a+\frac{b}{2})}\,, \tag{6}$$

$\Gamma(x)$ being the gamma function and $\mathcal{I} \equiv \int_0^L R(z)^{2-\frac{3b}{a}}\,dz$ remaining as the only factor depending on the particular geometry. Coefficients $\{a, b, \mathcal{C}\}$ depend on the blood damage model one is using; following [1], see Eq.(11), the averaged damaged hemoglobin count results into Eq. (17) in the main text.

Thus, Equation (6) provides a first approximation of the damaged hemoglobin count of any axisymmetric geometry, only requiring to compute the integral $\mathcal{I}$, which in most cases can be done analytically. Moreover, since the approximation consists in simplifying the actual flow by removing some disturbances, the obtained estimation shall be a lower limit of the actual value. Indeed, as mentioned in section Results and Discussion, this result underestimates blood damage in our particular study case by some $30-40\%$, mainly because we are neglecting any recirculating flow.

It is important to note here that the average has been computed over the actual damage and not over the still linear damage. The final result shall not be the same. Indeed, within this analytical framework one may explicitly show this fact by computing the two results, this is, Eq. (6): $\overline{D} \equiv \overline{(D_\ell)^a}$ against $\left(\overline{D_\ell}\right)^a$. Their ratio ends up being independent of the geometry,

$$\frac{\left(\overline{D_\ell}\right)^a}{\overline{D}} = \frac{(4a)^a/\,[2(b+2a)^a]}{\Gamma(2-a)\Gamma(\frac{b}{2}+1)/\Gamma(3-a+\frac{b}{2})} \overset{\text{Giersiepen}}{\simeq} 1.2521\ . \tag{7}$$

This means that computing averages over linear damage and then reverting the linearization overestimates the actual blood damage value by more than 25%. Figure S4 plots this ratio as contour lines on the parameter space of a and b hemolysis coefficients.

References

1. Giersiepen M, Wurzinger L (1990) Estimation of shear stress-related blood damage in heart valve prostheses–in vitro comparison of 25 aortic valves. The International Journal of Artificial Organs .
